# Reproducible candidate kinematic-electromyographic waveform markers of post-stroke gait from public multimodal waveform exports

**DOI:** 10.3389/fmedt.2026.1863908

**Published:** 2026-07-02

**Authors:** Rocco Salvatore Calabrò, Andrea Calderone, Fabrizio Sottile, Carmela Casella, Antonino Naro, Gokhan Ozkocak, Angelo Quartarone

**Affiliations:** 1 IRCCS Centro Neurolesi Bonino Pulejo, Messina, Italy; 2 Stroke Unit, AOU Policlinico Universitario, Messina, Italy; 3 Department of Physical Medicine and Rehabilitation, Faculty of Medicine, Istanbul Aydin University, Istanbul, Türkiye

**Keywords:** candidate digital biomarkers, gait analysis, gait asymmetry, kinematics, Neurorehabilitation, Stroke, surface electromyography, translational gait assessment

## Abstract

**Background:**

Instrumented gait analysis after stroke is informative but difficult to scale for routine rehabilitation use. This secondary analysis examined whether a spreadsheet-restricted subset of public sagittal kinematic and surface electromyography (sEMG) waveforms could yield clinically legible candidate waveform-derived markers and reproducible subject-level summaries.

**Methods:**

We analyzed a public multimodal gait dataset with 138 able-bodied adults and 50 adults with stroke. The workflow used only public spreadsheet exports and 11 domains shared across cohorts: four sagittal kinematic waveforms and seven normalized sEMG waveforms. Subject-level normative deviation and within-stroke asymmetry summaries were derived. Kinematics-only, sEMG-only, and combined kinematic-sEMG panels were compared using internal information-retention metrics within a nested benchmarking framework, not against an external clinical endpoint.

**Results:**

All kinematic domains were complete in both cohorts. Seven-channel sEMG completeness was lower, yielding 102 able-bodied controls, 43 paretic stroke sides, 44 non-paretic stroke sides, and 43 paired stroke participants for sEMG-containing complete-case benchmarking. Combined-panel deviation burden remained non-trivial bilaterally, with the largest median domain-level abnormalities in gastrocnemius activity and knee-angle waveforms. Relative to the combined internal reference panel, kinematics-only and sEMG-only reductions preserved substantial ranking information in 43 paired complete cases, with Pearson correlations of 0.865 and 0.875 and Spearman correlations of 0.839 and 0.884, respectively. Bootstrap intervals and sensitivity analyses indicated overlap between reduced panels and strongest robustness for kinematics-only summaries.

**Conclusion:**

Public spreadsheet waveform exports can support reproducible candidate gait markers, but reduced panels should be interpreted as internally benchmarked summaries rather than validated clinical biomarkers or prospective rehabilitation decision endpoints.

## 1 Introduction

Walking recovery remains a defining objective of stroke rehabilitation because the ability to move safely and efficiently underpins independence, participation, and access to community life. Recent work on gait measurement in neurorehabilitation has emphasized that clinically useful assessment must move beyond coarse bedside observation and must capture how patients actually walk, not only whether they can walk (1). Kinematic synthesis studies likewise show that post-stroke ambulation cannot be understood through speed alone, because meaningful deficits emerge in joint motion, segmental coordination, and interlimb timing that remain clinically relevant even in ambulatory survivors (2). This broader perspective is especially important because gait asymmetry is not a marginal phenomenon after stroke. It is a core feature of hemiparetic locomotion and is shaped by motor impairment, sensory loss, strength deficits, and compensatory strategies that may persist long after basic walking has returned (3).

The increasing use of portable sensing technologies has created a strong expectation that gait assessment can be made more scalable, more objective, and more responsive to real clinical needs. Systematic reviews of post-stroke wearable assessment have confirmed promising measurement properties for several platforms, but they have also highlighted uneven methodological quality, variable construct definitions, and limited agreement across devices and analytic pipelines (4). Similar caution emerges from studies of inertial measurement units, which can perform well in selected contexts but still require careful validation before their outputs can be treated as interchangeable with higher-complexity laboratory measures (5). At the same time, routine implementation remains far from mature. Even when clinicians value wearable monitoring, day-to-day adoption in stroke care is still constrained by workflow burden, uncertainty about interpretation, and the absence of compact outputs that are both technically sound and clinically legible (6). Comparable concerns have also been raised for instrumented walkways and related systems, where reliability can be good, yet transferability across settings and protocols cannot be taken for granted (7).

For that reason, instrumented gait analysis remains a crucial reference standard in neurorehabilitation. Its value lies not only in measurement precision, but also in its capacity to expose mechanisms that are clinically hidden when walking is summarized by a handful of spatiotemporal metrics. Classic rehabilitation literature has long argued that gait analysis becomes useful when it links observed movement patterns to underlying impairments and to the compensatory solutions adopted by patients (8). That argument still holds in stroke. Hemiparetic walking is characterized by a complex reorganization of propulsion, limb advancement, pelvic motion, and trunk control, rather than by a single uniform deficit (9). Seemingly simple patterns such as hip hiking or circumduction are therefore not trivial descriptors. They represent quantifiable movement solutions that may compensate for weakness, stiffness, or impaired limb clearance, and they often coexist with multiple other deviations within the same individual (10).

This heterogeneity is one reason why asymmetry deserves sustained attention. Community-ambulating stroke survivors frequently continue to display marked interlimb asymmetry despite achieving functional walking, which means that conventional success indicators can obscure abnormal biomechanical organization (11). Recent work has further shown that even when propulsion asymmetry is experimentally modified, the broader asymmetry profile may change only modestly, again underscoring that post-stroke gait cannot be reduced to a single variable or a single mechanism (12). In parallel, the rehabilitation landscape is increasingly populated by wearable devices, exoskeletons, robot-assisted training, and biofeedback-enabled interventions, all of which need meaningful gait markers if they are to support personalized monitoring rather than merely generate more data (13). Trials and meta-analyses of activity-focused walking interventions and technology-assisted gait rehabilitation continue to show that intensity and technological support can improve walking outcomes, yet they do not resolve the parallel question of which movement signatures should be tracked when recovery and compensation coexist (14–16).

This unresolved measurement problem helps explain the growing interest in data-driven rehabilitation. Artificial intelligence and related analytic approaches are now being explored throughout adult stroke recovery, including prediction, monitoring, classification, and therapy personalization (17). Broader rehabilitation literature shows a similar trend, with machine learning increasingly used to process high-dimensional movement data that are difficult to summarize by manual inspection alone (18). In the specific field of gait, automatic classification systems and machine-learning pipelines have grown rapidly, particularly in stroke and cerebral palsy, but recent reviews repeatedly note that translational value depends less on technical novelty than on feature relevance, interpretability, and robustness to real-world clinical constraints (19, 20).

These considerations motivated the present study. Rather than testing another high-dimensional classifier, we asked whether a restricted set of exported sagittal kinematic and surface electromyography (sEMG) waveforms could be transformed into interpretable subject-level candidate waveform-derived markers that preserve useful information after signal-space reduction. The public dataset used here offers a transparent setting for this question because it contains able-bodied adults and adults with stroke processed within the same experimental framework, while the spreadsheet exports permit a reproducible workflow without relying on inaccessible or proprietary data structures. The conceptual goal was not to validate clinical biomarkers or replace comprehensive gait-laboratory assessment, but to internally benchmark how much interpretable waveform information is retained when a multimodal representation is reduced to parsimonious kinematic-sEMG panels.

## 2 Materials and methods

### 2.1 Study design and public data source

This investigation was conducted as a secondary analysis of a deidentified public dataset and was written in line with the general reporting logic of the Strengthening the Reporting of Observational Studies in Epidemiology and Reporting of studies Conducted using Observational Routinely-collected health Data statements (21, 22). The source resource was the multimodal gait dataset published by Van Criekinge and colleagues, which includes able-bodied adults assessed across the adult life span together with adults with stroke studied within the same laboratory ecosystem (23). The objective was translational rather than predictive. We sought to determine whether a restricted, fully shareable waveform space could be summarized as subject-level candidate waveform-derived markers that remain interpretable after signal reduction, while avoiding unsupported claims about diagnosis, prognosis, treatment response, clinical validity, or replacement of comprehensive gait-laboratory assessment.

The current workflow was deliberately constrained to the public spreadsheet exports and companion documentation rather than the full post-processed MATLAB structures. This design choice shaped the entire work. It prevented re-entry into hidden trial-level structures, ensured that every derived quantity could be reconstructed from the publicly available repository files, and aligned the analysis with the practical reality of reusable public exports. The benchmark in this study was therefore an internal one. Reduced signal panels were evaluated against a combined shared kinematic-sEMG panel rather than against an external clinical endpoint, diagnostic label, prospective outcome, or device measurement. Because the combined kinematic-sEMG panel contains the same domains that appear in the reduced panels, these comparisons should be interpreted as internal information-retention benchmarking within a nested feature space rather than as independent validation.

### 2.2 Available files, participants, and analysis-ready sample

Two spreadsheet exports constituted the primary analytic input: MAT_normalizedData_AbleBodiedAdults_v06-03-23.xlsx for able-bodied adults and MAT_normalizedData_PostStrokeAdults_v27-02-23.xlsx for adults with stroke. The able-bodied workbook contained a readme sheet and 138 participant sheets, whereas the stroke workbook contained a readme sheet and 50 participant sheets. In both workbooks, each participant sheet stored 1001 time-normalized samples covering 0%–100% of the gait cycle. The present work did not reprocess the source C3D files, force-plate signals, or full MATLAB structures. Instead, it treated the spreadsheets as the analysis-ready layer of the public resource. The analysis-ready cohort structure, file provenance, and domain restriction are summarized in Table 1.

**Table 1:** Cohort structure and analysis-ready data domains.

Item	Able-bodied export	Stroke export	Interpretive note
Public cohort size	138 able-bodied adults	50 stroke survivors	Cohort counts from the public spreadsheet exports
Age range reported in data descriptor	21–86 years	19–85 years	Used only to describe the source cohort; age was not re-derived from the uploaded Excel exports.
Spreadsheet file analyzed in this work	MAT_normalizedData_AbleBodiedAdults_v06-03-23.xlsx	MAT_normalizedData_PostStrokeAdults_v27-02-23.xlsx	Current manuscript deliberately relies on the spreadsheet exports rather than the full MAT structures.
Subject-level worksheet structure	ReadMe + 138 subject sheets (Sub01–Sub138)	ReadMe + 50 subject sheets (Sub01–Sub50)	Each subject sheet contains one time-normalized waveform per exported variable.
Time-normalized waveform length	1001 samples per variable	1001 samples per variable	All nonmissing exported waveforms had full 1001-point coverage.
Shared sagittal kinematic domains used here	Ankle, knee, hip, and pelvis angles	Paretic and non-paretic ankle, knee, hip, and pelvis angles	These four domains define the kinematics-only panel.
Shared normalized sEMG domains used here	GASnorm, RFnorm, VLnorm, BFnorm, STnorm, TAnorm, ERSnorm	Paretic and non-paretic GASnorm, RFnorm, VLnorm, BFnorm, STnorm, TAnorm, ERSnorm	These seven domains define the sEMG-only panel.
Domainwise complete shared kinematic waveforms	138/138 for each of the four shared kinematic domains	50/50 paretic and 50/50 non-paretic for each of the four shared kinematic domains	No partial within-waveform missingness was observed.
Domainwise complete shared sEMG waveforms	106–109/138 depending on muscle	43–46/50 paretic and 44–46/50 non-paretic depending on muscle	sEMG missingness was all-or-none at the waveform level within each domain.
Complete-case size for the full 11-domain shared panel	102/138	43/50 paretic and 44/50 non-paretic	This rule was used for the primary combined-panel benchmarking analyses.
Analysis-ready paired asymmetry sample	Not applicable in the uploaded export because control sides were not preserved separately	43/50 for the full 11-domain combined asymmetry panel; 50/50 for the four-domain kinematic asymmetry panel	Asymmetry analyses were therefore performed only within the stroke cohort.
Reduced-panel paired benchmark	Not applicable	43 paired stroke participants	Distinct from 43 paretic/44 non-paretic side-specific counts and 46-per-side ERS availability.

The first two data columns report the exact public exports that were used for the present workflow. The final column explains why each item matters for the current work and clarifies where the analysis was intentionally restricted.

sEMG, surface electromyography; GRF, ground reaction force; MAT, MATLAB structure; GAS, gastrocnemius; RF, rectus femoris; VL, vastus lateralis; BF, biceps femoris; ST, semitendinosus; TA, tibialis anterior; ERS, erector spinae.

No participant-level exclusions were imposed beyond waveform availability within the selected shared domains. For descriptive context only, the data descriptor reports an age range of 21–86 years in the able-bodied cohort and 19–85 years in the stroke cohort (23). The descriptor also states that the original acquisition pipeline used conventional laboratory gait analysis and standardized sEMG placement practices (24, 25). In the source processing, rectified sEMG was band-pass filtered, smoothed to a linear envelope, and normalized to the maximum value across available strides for each muscle, so the current sEMG variables represent normalized waveform amplitude and timing rather than raw voltage (23). The spreadsheet-restricted layer did not contain a complete participant-level demographic or clinical table linked to sEMG completeness; therefore, sEMG-complete and sEMG-incomplete subsets could not be compared for selection bias within this workflow. All study-specific calculations reported here were performed from the exported subject-level waveforms already contained in the spreadsheets.

### 2.3 Signal domains and variable selection

The analytic space was intentionally limited to domains that were verifiably available in both cohorts and therefore comparable without asymmetrical information content. This yielded an eleven-domain shared space consisting of four sagittal kinematic waveforms and seven normalized sEMG waveforms. The kinematic domains were ankle, knee, hip, and pelvis angles. The normalized sEMG domains were gastrocnemius, rectus femoris, vastus lateralis, biceps femoris, semitendinosus, tibialis anterior, and erector spinae. In the able-bodied workbook, these domains were represented as one participant-level waveform per domain. In the stroke workbook, each domain was represented separately for the paretic and non-paretic side.

This restriction served two methodological purposes. First, it prevented the stroke cohort from being compared with an able-bodied feature space enriched by kinetics or ground-reaction-force variables that were not equivalently exported in the stroke workbook. Second, it kept the translational target aligned with data types that are conceptually closer to reduced sensing implementations. The unit of analysis was the participant-level waveform, not the stride. Consequently, all downstream summaries describe average waveform behavior after prior stride processing performed in the public dataset, rather than stride-to-stride variability or event-by-event fluctuation.

### 2.4 Candidate digital biomarker framework

A normative reference framework was constructed separately for each of the eleven shared domains using the able-bodied cohort. For every time-normalized sample point, the primary workflow computed the pointwise mean and standard deviation, while a robust companion framework used the pointwise median and median absolute deviation-derived dispersion estimate. Because the spreadsheet exports did not provide an age-, sex-, walking-speed-, or side-matched control structure for each stroke participant, this reference was deliberately broad and public rather than a matched clinical normative sample. Deviation scores for the stroke cohort were then derived side by side from the paretic and non-paretic waveforms. This strategy was conceptually related to established traditions of compact gait summary scoring and to stroke-specific work on gait symmetry and asymmetry interpretation (26–30). The operational definitions of all candidate marker families used are summarized in Table 2.

**Table 2:** Operational definitions of the candidate waveform-derived markers.

Biomarker	Analytic family	Applicable domains	Operational definition	Interpretive meaning	Direction of more abnormality
Mean absolute standardized deviation (MASD)	Normative deviation	All shared kinematic and normalized sEMG domains	MASD_*d*, *s* = mean_*t*|(*x*_*d*, *s*(*t*) − *μ*_*d*(*t*))/*σ*_*d*(*t*)|	Average standardized abnormality burden across the full normalized gait cycle for one domain and one stroke side	Higher values indicate greater abnormality
Root mean square standardized deviation (RMSD)	Normative deviation	All shared kinematic and normalized sEMG domains	RMSD_*d*, *s* = sqrt(mean_*t* [((*x*_*d*, *s*(*t*) − *μ*_*d*(*t*))/*σ*_*d*(*t*))^2^])	Deviation summary that gives extra weight to larger departures from the reference waveform	Higher values indicate greater abnormality
Peak absolute standardized deviation (PASD)	Normative deviation	All shared kinematic and normalized sEMG domains	PASD_*d*, *s* = max_*t* |(*x*_*d*, *s*(*t*) − *μ*_*d*(*t*))/*σ*_*d*(*t*)|	Most extreme standardized departure observed at any time point of the normalized cycle	Higher values indicate greater abnormality
Time outside the 95% normative corridor (TONC95)	Normative deviation	All shared kinematic and normalized sEMG domains	TONC95_*d*, *s* = 100 × mean_*t I*[*x*_*d*,*s*(*t*) < *μ*_*d*(*t*) − 1.96*σ*_*d*(*t*) or *x*_*d*,*s*(*t*) > *μ*_*d*(*t*) + 1.96*σ*_*d*(*t*)]	Percentage of the gait cycle spent outside the pointwise reference corridor	Higher values indicate greater abnormality
Normative waveform correlation (NWC)	Normative deviation	All shared kinematic and normalized sEMG domains	NWC_*d*,*s* = corr(*x*_*d*,*s*(*t*), *μ*_*d*(*t*))	Shape similarity between the observed waveform and the normative reference waveform	Lower values indicate greater abnormality
Mean absolute standardized asymmetry (MASA)	Within-stroke asymmetry	All shared kinematic and normalized sEMG domains with both sides available	MASA_*d* = mean_*t* |(*x*_*d*,*P*(*t*) − *x*_*d*,*N*(*t*))/*σ*_*d*(*t*)|	Average standardized interlimb discrepancy across the normalized gait cycle	Higher values indicate greater asymmetry
Root mean square standardized asymmetry (RMSA)	Within-stroke asymmetry	All shared kinematic and normalized sEMG domains with both sides available	RMSA_*d* = sqrt(mean_*t* [((*x*_*d*,*P*(*t*) − *x*_*d*,*N*(*t*))/*σ*_*d*(*t*))^2^])	Asymmetry summary that emphasizes larger interlimb differences	Higher values indicate greater asymmetry
Side waveform correlation (SWC)	Within-stroke asymmetry	All shared kinematic and normalized sEMG domains with both sides available	SWC_*d* = corr(*x*_*d*,*P*(*t*), *x*_*d*,*N*(*t*))	Shape similarity between paretic and non-paretic waveforms within the same participant	Lower values indicate greater asymmetry
Absolute sEMG centroid-timing asymmetry (AECTA)	Within-stroke asymmetry	Normalized sEMG domains only	AECTA_*m* = |CT_*m*,*P* − CT_*m*,*N*|, where CT = Σ[*t* × sEMG(*t*)]/Σ[sEMG(*t*)]	Difference in the temporal center of mass of normalized muscle activity between the two sides, expressed as percent gait cycle	Higher values indicate greater asymmetry

Each row defines one marker family used in the current manuscript framework. The operational-definition column gives the exact mathematical object that should be computed from the public waveforms, while the last column states the clinically interpretable direction of worse abnormality or greater asymmetry. mu_*d*(*t*) and sigma_*d*(*t*) denote the pointwise able-bodied reference mean and standard deviation for domain d, x_d,s(t) denotes the observed waveform for side s, and P/N denote paretic and non-paretic sides in the stroke cohort.

MASD, mean absolute standardized deviation; RMSD, root mean square standardized deviation; PASD, peak absolute standardized deviation; TONC95, time outside the 95% normative corridor; NWC, normative waveform correlation; MASA, mean absolute standardized asymmetry; RMSA, root mean square standardized asymmetry; SWC, side waveform correlation; AECTA, absolute sEMG centroid-timing asymmetry; sEMG, surface electromyography.

Two complementary candidate marker layers were retained. The first layer quantified normative deviation relative to the able-bodied reference using mean absolute standardized deviation (MASD), root mean square standardized deviation, peak absolute standardized deviation, time outside the 95% normative corridor, and normative waveform correlation. MASD was selected as the primary composite burden metric because it provides a directionally interpretable full-cycle average, is less dominated by single extreme samples than peak-based summaries, and remains suitable for subject-level panel aggregation. The second layer quantified within-stroke asymmetry using mean absolute standardized asymmetry, root mean square standardized asymmetry, side waveform correlation, and absolute sEMG centroid-timing asymmetry. These companion metrics describe complementary properties of the same waveform space, including large excursions, time outside the reference corridor, shape similarity, interlimb discrepancy, and sEMG timing. Normative deviation and asymmetry were treated as non-interchangeable constructs. A participant could deviate from the able-bodied reference on both sides while remaining relatively symmetric, or conversely show marked between-side asymmetry despite only moderate global abnormality.

### 2.5 Reduced signal panels and benchmarking strategy

Three reduced signal panels were prespecified. The kinematics-only panel contained the four sagittal angle domains. The sEMG-only panel contained the seven normalized sEMG domains. The combined kinematic-sEMG panel contained all eleven shared domains and was treated as the internal reference representation for signal-reduction benchmarking, not as an external gold standard. Complete-case rules were applied separately within panel families: the kinematics-only panel retained all 50 stroke participants, whereas the sEMG-only and combined panels inherited the more restrictive completeness of the normalized sEMG exports. Side-specific combined deviation scores were available for 43 paretic and 44 non-paretic stroke sides, while the paired complete-case sample used for asymmetry and reduced-panel benchmarking included 43 stroke participants. The panel definitions, translational rationale, and complete-case logic are summarized in Table 3.

**Table 3:** Reduced signal panels and benchmarking framework.

Panel	Included domains	Primary translational rationale	Primary benchmarking score	Complete-case rule	Analysis-ready sample size	Prespecified sensitivity checks
Kinematics-only panel	AnkleAngles, KneeAngles, HipAngles, PelvisAngles	Motion-only representation that remains closest to simplified clinical waveform assessment while preserving the major sagittal lower-limb and pelvic domains shared by both groups	Panel mean of domain-level MASD	All four kinematic domains required	Controls 138/138; stroke paretic 50/50; stroke non-paretic 50/50; paired asymmetry 50/50	Robust reference dispersion and broad-window representation
sEMG-only panel	GASnorm, RFnorm, VLnorm, BFnorm, STnorm, TAnorm, ERSnorm	Muscle-activity representation that isolates neural activation timing and amplitude structure without kinematic information	Panel mean of domain-level MASD; asymmetry summaries reported separately	All seven normalized sEMG domains required for the primary complete-case score	Controls 102/138 complete across all seven domains; stroke paretic 43/50; stroke non-paretic 44/50; paired asymmetry 43/50	Robust reference dispersion and ERS-exclusion sensitivity analysis
Combined kinematic-sEMG panel	All four shared kinematic domains plus all seven shared normalized sEMG domains	Nested internal multimodal reference panel for benchmarking reduced signal strategies in the present manuscript; not an external gold standard	Panel mean of domain-level MASD with companion asymmetry composite	All eleven domains required for the primary complete-case score	Controls 102/138; stroke paretic 43/50; stroke non-paretic 44/50; paired asymmetry 43/50	Robust reference dispersion, broad-window representation, ERS-exclusion, and available-case comparison

This table defines the three panel families examined in the present analysis and clarifies their composition, intended analytical role, and benchmarking strategy. The sample-size column refers to analysis-ready complete cases in the available post-processed exports. The Figure 3 reduced-panel benchmark used the 43 paired stroke participants complete for both sides across the kinematics-only, sEMG-only, and combined panels.

sEMG, surface electromyography; MASD, mean absolute standardized deviation; ERS, erector spinae.

Panel reduction was evaluated through information-retention benchmarking rather than outcome prediction. This decision reflects the absence of a fully verifiable external clinical endpoint in the uploaded spreadsheets. Panel scores were defined as the arithmetic mean of domain-level MASD values across the domains belonging to a given panel. Equal domain weighting was chosen *a priori* as a transparent, non-optimized aggregation rule to avoid data-driven weights or endpoint-dependent overfitting; it should not be interpreted as evidence that all kinematic and sEMG domains have identical physiological importance, reliability, or clinical salience. The current agreement metrics quantify information retention relative to the internal multimodal reference representation rather than device validity, clinical responsiveness, or prognostic performance. The reduced panels were therefore compared with the combined panel using subject-level composite deviation summaries, rank-based agreement, top-burden overlap, quartile agreement, and standardized root mean square error. Because the combined kinematic-sEMG panel contains the same domains that appear in the reduced panels, these comparisons should be interpreted as internal information-retention benchmarking within a nested feature space rather than as independent validation.

### 2.6 Statistical analysis and sensitivity analyses

The analysis was performed at the participant level using deterministic scripts operating directly on the spreadsheet XML files. The computational workflow was implemented in Python 3.13.5 using NumPy 2.3.5, SciPy 1.17.0, lxml 6.1.0, and matplotlib 3.10.8 for figure rendering. No stochastic model fitting was used. Because the primary outputs were waveform-derived summaries rather than clinical trial endpoints, the statistical emphasis was descriptive and robustness-oriented. Continuous summaries are therefore reported mainly as medians with interquartile ranges, together with agreement coefficients and score-difference metrics for the benchmarking contrasts. Nonparametric bootstrap intervals were added for the main reduced-panel agreement metrics using 5,000 resamples and fixed seed 1,863,908. These intervals were treated as descriptive uncertainty summaries; no formal hypothesis testing against an external endpoint was prespecified, and no p values were generated for the primary results.

Sensitivity analyses were specified *a priori* to test how strongly the candidate marker architecture depended on particular representational choices. Four deviation-oriented contrasts were examined: replacement of mean and standard deviation with robust reference dispersion, exclusion of the erector spinae channel from sEMG-containing panels, reduction of each waveform to five broad 20% gait-cycle windows, and comparison of complete-case versus available-case scoring. The 20% windows were used as a pragmatic phase-coarsening sensitivity analysis that retained clinical interpretability while testing whether the full-cycle burden summary depended on local waveform detail. A fifth sensitivity contrast examined amplitude-normalized asymmetry. For each sensitivity scenario, the primary outputs were rank agreement with the main analysis, absolute score differences, and quartile reclassification. The complete-case specification was retained as the primary analysis because it preserved identical panel composition across participants, whereas the available-case rule changes the contributing domain set across subjects and was therefore treated only as a sensitivity analysis that quantifies the practical cost of strict multimodal completeness. The full sensitivity design is summarized in Table 4.

**Table 4:** Prespecified sensitivity and robustness analyses.

Sensitivity analysis	Applied to	Methodological change	Primary comparison metric	Why it matters
Robust normative dispersion	Deviation scores for all panels	Replace pointwise able-bodied mean and standard deviation with pointwise median and MAD-derived robust dispersion	Spearman agreement with the primary panel score, absolute score difference, and quartile reclassification	Tests whether the biomarker architecture depends excessively on non-robust reference scaling
ERS-exclusion analysis	sEMG-only and combined panels; asymmetry sEMG summaries	Repeat the workflow after removing ERSnorm from the panel definition	Change in panel scores and rank agreement relative to the primary analysis	Tests whether the trunk channel disproportionately drives panel behavior
Broad-window representation	Deviation scores for all panels	Reduce each waveform to five broad 20% gait-cycle windows and recompute the primary deviation summary from the window means	Spearman agreement with the full-cycle score and absolute score difference	Tests whether the conclusions remain stable when local waveform detail is compressed
Complete-case versus available-case comparison	Combined panel only	Compare the primary complete-case panel score with a secondary combined score that allows kinematic-only contribution when all four shared kinematic domains are present	Overlap agreement plus number of additional subjects recovered in the secondary analysis	Quantifies the practical cost of strict multimodal completeness without replacing the primary complete-case definition
Amplitude-normalized asymmetry	Asymmetry scores for all panels	Recompute side-difference summaries after within-waveform centering and scaling of each limb trace before subtraction	Spearman agreement with the primary asymmetry score and absolute score difference	Tests whether the asymmetry ranking is dominated by amplitude rather than waveform shape

Each row states one executable robustness contrast for the current MedTech workflow and can be matched directly to the companion sensitivity-results supplementary table. Bootstrap uncertainty for the reduced-panel internal agreement metrics is reported separately in Supplementary Material 5.

MAD, median absolute deviation; ERS, erector spinae; sEMG, surface electromyography.

### 2.7 Reproducibility, transparency, and ethics

**Figure 1:**
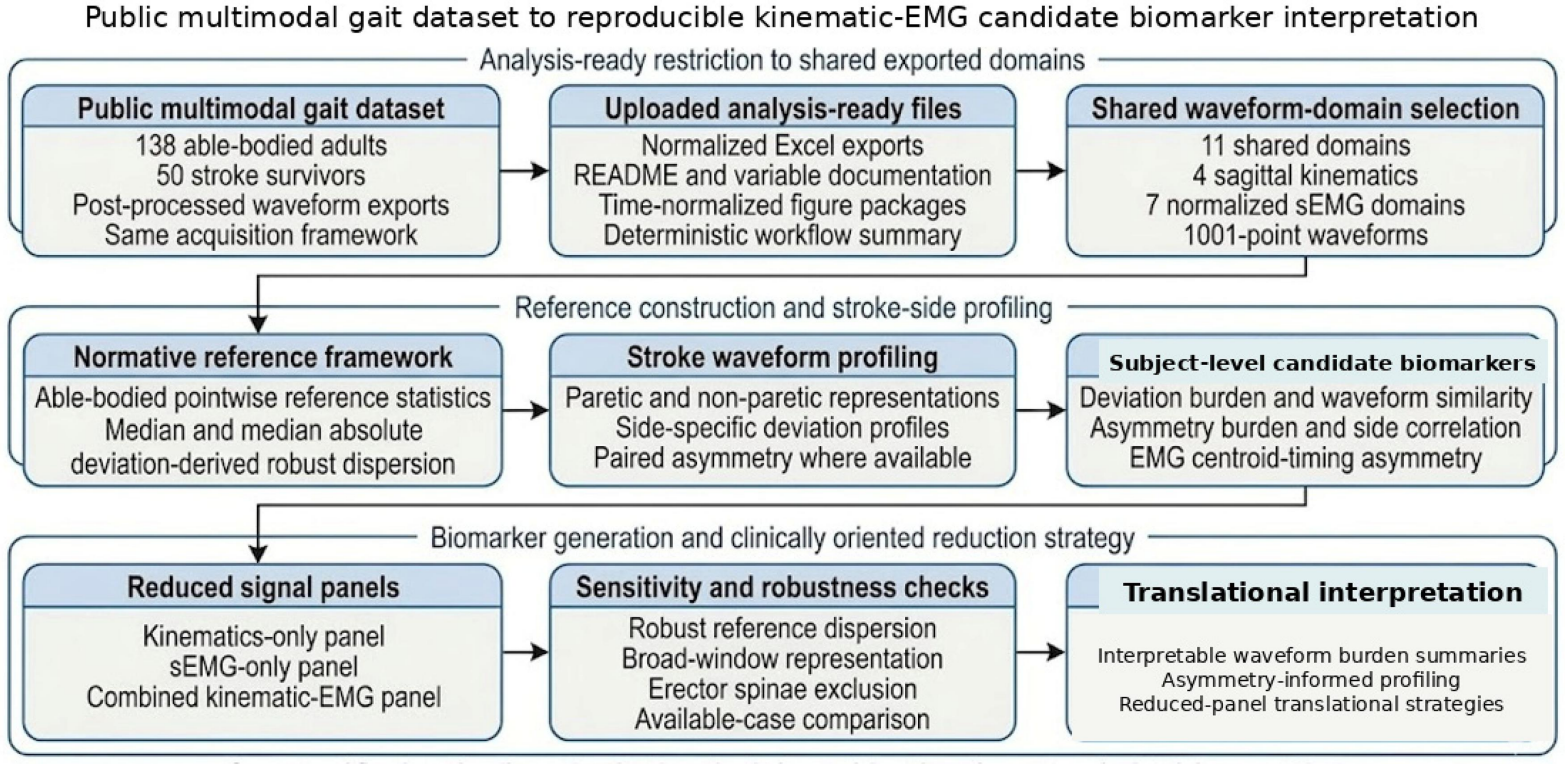
Study design and translational workflow.

All transformations were designed to be reproducible from the uploaded exports without proprietary preprocessing. The workflow consisted of importing subject sheets, restricting the analysis to the eleven shared domains, constructing pointwise able-bodied reference statistics, computing side-specific deviation metrics, deriving within-stroke asymmetry metrics, aggregating panel-level scores under prespecified complete-case rules, and computing fixed-seed bootstrap intervals for the main agreement metrics. The overall workflow is summarized in Figure 1. The exact input filenames, column mappings, mathematical definitions, sensitivity outputs, bootstrap seed, and generated benchmark tables are provided in Supplementary Materials 1–5 and in the accompanying reproducibility package.

The original source dataset involved human participants and was collected with local ethics approval and written informed consent, as reported in the data descriptor. The present study was a secondary analysis of publicly available de-identified human participant data and did not involve new recruitment, participant contact, intervention, identifiable information, or additional experimental procedures. The ethical emphasis of the current workflow therefore lay in transparency, reproducibility, and restrained interpretation. All analyses were confined to what could be directly supported by the uploaded files, and all interpretations were framed as internally benchmarked translational summaries rather than as validated clinical-device outputs.

Artificial intelligence was used only as a visual-support tool during preparation and refinement of the manuscript figures, including graphical organization, layout optimization, and visual clarity checks. ChatGPT (OpenAI; version available at the time of manuscript preparation) was not used to generate, alter, or analyze the underlying dataset, to perform statistical analyses, to create numerical results, or to determine the scientific interpretation or conclusions. Numerical values in the figures were generated from the reproducible analysis workflow and were checked by the authors. All figure content, labels, analytical outputs, and final visual representations were reviewed and approved by the authors, who remain fully responsible for the integrity and accuracy of the submitted material.

## 3 Results

### 3.1 Analysis-ready shared signal space

The spreadsheet-restricted workflow yielded a fully comparable eleven-domain analytic space composed of four sagittal kinematic waveforms and seven normalized sEMG waveforms. All four kinematic domains were complete in all 138 able-bodied participants and in both sides of all 50 participants with stroke. sEMG completeness was lower but transparent. In the able-bodied cohort, complete individual-domain sEMG waveforms were available for 106–109 participants depending on the channel, and 102 participants had complete data across all seven normalized sEMG domains. In the stroke cohort, the first six normalized sEMG channels were complete in 43 paretic and 44 non-paretic sides, whereas erector spinae was available in 46 participants per side. The latter value reflects ERS-only availability and should not be confused with the complete seven-channel sEMG or eleven-domain combined panel. Consequently, the primary combined and sEMG-based complete-case panels contained 43 paretic, 44 non-paretic, and 43 paired participants for asymmetry and reduced-panel benchmarking, whereas the kinematics-only panel retained all 50 stroke participants.

These completeness patterns shaped the panel-level summaries. For deviation burden, the kinematics-only panel showed median composite scores of 1.29 on the paretic side and 1.38 on the non-paretic side. The corresponding sEMG-only medians were 1.54 and 1.47, and the combined-panel medians were 1.49 and 1.38. Thus, abnormality burden was not confined to the paretic side. The asymmetry layer added a different perspective. Median asymmetry composite scores were 0.88 for the kinematics-only panel, 1.26 for the sEMG-only panel, and 1.22 for the combined panel, indicating that sEMG-containing summaries captured more between-side disequilibrium than kinematics alone.

At the domain level, gastrocnemius and knee-angle waveforms showed the largest median deviation burdens on both sides, followed by erector spinae and selected quadriceps channels. Pelvis and hip angles tended to show lower burdens. For the asymmetry summaries, the largest median domain-level scores were observed for gastrocnemius, semitendinosus, knee angle, and erector spinae, whereas pelvis angle remained the least asymmetric domain. Overall, the exported waveform space retained both side-specific abnormality and between-side imbalance rather than collapsing them into a single movement signal.

### 3.2 Representative profiles and information retention after panel reduction

**Figure 2:**
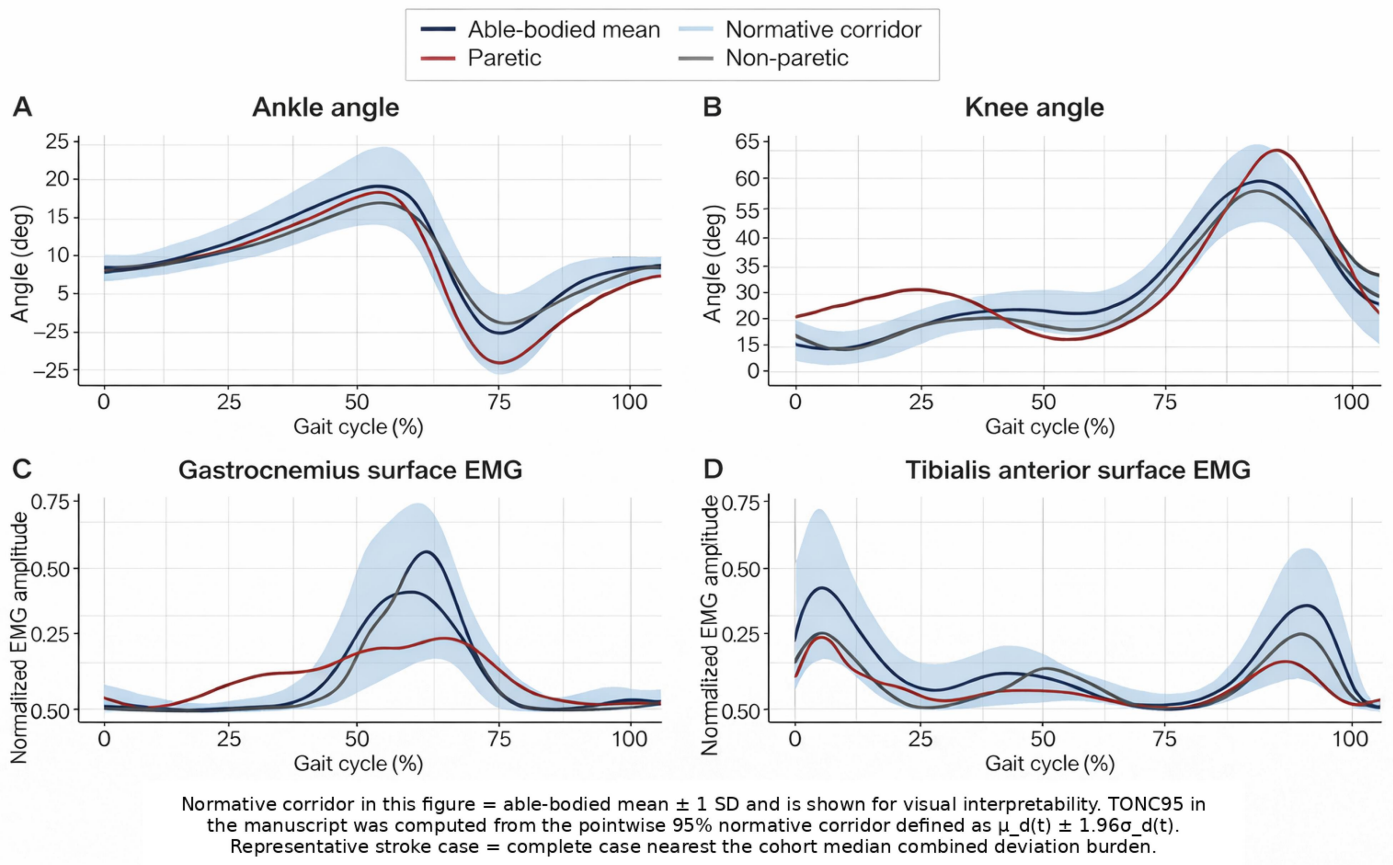
Representative normative corridor and stroke deviation profiles across shared kinematic and sEMG domains.

A representative visualization of the workflow output is shown in Figure 2. The displayed case was selected as the complete case nearest the cohort median combined deviation burden and was used for visual contextualization rather than formal inference. For visual legibility, the shaded corridor in Figure 2 represents the able-bodied mean ± 1 standard deviation, whereas the TONC95 candidate marker was computed from the pointwise 95% normative corridor defined as mu_*d*(*t*) ± 1.96 sigma_*d*(*t*). The figure is therefore illustrative of deviation structure rather than a direct graphical rendering of TONC95. Even with this cautious framing, the figure illustrates the qualitative logic of the candidate marker architecture. The ankle panel shows a more abnormal late-stance and early-swing profile on the paretic side than on the non-paretic side. The knee panel shows a delayed and accentuated flexion pattern relative to the able-bodied reference corridor. The gastrocnemius and tibialis anterior panels show disturbed activation timing and broader departures from the normative corridor than would be visible from discrete gait events alone.

**Figure 3:**
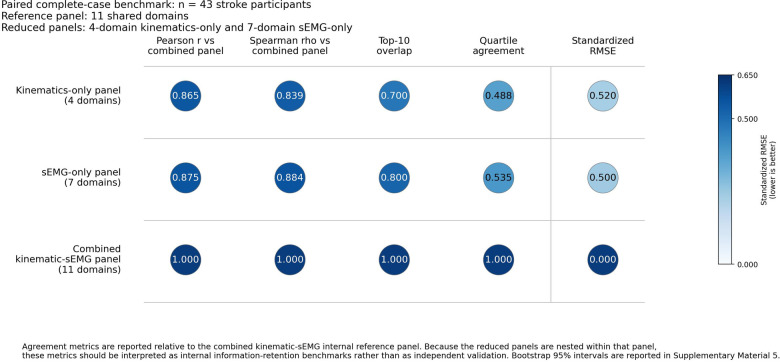
Information retention of reduced signal panels relative to the combined kinematic-sEMG internal reference panel.

Internal benchmarking showed that neither reduced panel fully reproduced the combined kinematic-sEMG representation, although both retained substantial ranking information. Because the combined panel contains the domains of the reduced panels, these metrics quantify internal information retention within the current dataset rather than independent validation. In the 43 paired complete cases used for Figure 3, the kinematics-only panel achieved a Pearson correlation of 0.865 and a Spearman correlation of 0.839 relative to the combined panel, whereas the sEMG-only panel achieved a Pearson correlation of 0.875 and a Spearman correlation of 0.884. The corresponding 95% bootstrap intervals were 0.791–0.914 and 0.719–0.903 for the kinematics-only Pearson and Spearman estimates, and 0.797–0.931 and 0.775–0.936 for the sEMG-only Pearson and Spearman estimates. Top-10 overlap was 0.700 for kinematics-only and 0.800 for sEMG-only, whereas quartile agreement remained moderate at 0.488 and 0.535. Standardized root mean square error likewise remained non-trivial at 0.520 and 0.500, respectively. Full bootstrap intervals for all Figure 3 metrics are reported in Supplementary Material 5.

### 3.3 Sensitivity analyses and reproducibility outputs

Robustness analyses showed that stability depended on both panel content and score definition. Replacing the pointwise mean and standard deviation with robust reference scaling preserved rank ordering very strongly, especially for the kinematics-only panel, where Spearman agreement reached 0.995 for the non-paretic side and 0.996 for the paretic side. Even so, quartile reclassification remained 14.0%–20.0% for kinematics. The corresponding robust-scaling contrasts for the combined and sEMG-only deviation panels also preserved high rank agreement, with rho values between 0.979 and 0.991, but quartile movement was materially larger, ranging from 55.8% to 63.6% in the combined panel and from 60.5% to 63.6% in the sEMG-only panel. Compression of the waveforms into broad 20% gait-cycle windows produced a similar pattern, with the kinematics-only panel again showing the smallest perturbation.

Removal of the erector spinae channel produced smaller changes than either robust scaling or broad-window compression. For deviation scores, combined-panel rank agreement remained between 0.965 and 0.991 with quartile reclassification between 11.6% and 18.2%, while the sEMG-only panel showed rho values between 0.947 and 0.970 with quartile reclassification between 23.3% and 31.8%. Available-case rescoring reproduced the overlapping complete-case scores exactly, with rho values of 1.000, zero median score difference, and zero quartile reclassification. This occurred because the secondary rule added cases only in the combined panel and did not alter scores for overlapping participants, whose primary values were already defined on the full eleven-domain set. By contrast, amplitude-normalized asymmetry was the least stable contrast, with rho values of 0.838 for the combined panel, 0.748 for the sEMG-only panel, and 0.778 for the kinematics-only panel, together with quartile reclassification between 58.0% and 74.4%.

The companion materials make the workflow auditable at several levels. The exact column-to-domain mapping used in the current workflow is provided in Supplementary Material 1. Domain-level availability and quality checks are collated in Supplementary Material 2. Full mathematical definitions of the deviation and asymmetry candidate markers used in the current workflow are compiled in Supplementary Material 3. The full results of the prespecified sensitivity analyses are reproduced in Supplementary Material 4. Bootstrap intervals, sample definitions for Figure 3, input filenames, generated output tables, and the executable reproducibility script are provided in Supplementary Material 5 and the accompanying reproducibility package.

## 4 Discussion

### 4.1 Principal findings and translational interpretation

This study examined whether a spreadsheet-restricted subset of a public multimodal gait dataset could support clinically legible candidate waveform-derived markers after signal-space reduction. Three findings stand out. First, the shared eleven-domain waveform space was sufficient to capture post-stroke abnormality on both the paretic and non-paretic side relative to a broad public able-bodied reference. Second, normative deviation and asymmetry provided complementary rather than redundant information. Third, kinematics-only and sEMG-only reductions retained substantial but incomplete information relative to the combined internal panel. In practical terms, a reduced panel may still identify participants with relatively high waveform burden, yet it cannot be assumed to reproduce the finer ordering of a multimodal representation. Recent reviews of wearable and biofeedback-enabled stroke rehabilitation have similarly argued that clinically useful digital markers must balance scalability with interpretability rather than simply maximize data volume or algorithmic complexity (31, 32). Longitudinal monitoring studies and early outcome work using wearables likewise show promise, but they also underscore that translational usefulness depends on how the underlying signals are summarized, visualized, and related to recognizable clinical constructs (33, 34). Accordingly, the present contribution is best understood as a medtech data-analytics workflow for interpretable signal reduction rather than as a clinical biomarker-validation or device-validation study.

The present results refine that broader literature by showing that signal reduction is not a binary success-or-failure problem. Both reduced panels preserved broad participant ranking, yet neither behaved as an interchangeable substitute for the combined panel. The similar benchmarking profiles of the kinematics-only and sEMG-only panels, together with overlapping bootstrap intervals, suggest that motion and muscle-activation waveforms each encode a different but related slice of post-stroke gait organization. Because these benchmarks were computed against a nested combined panel rather than an independent external criterion, they should be read as evidence of partial information retention and not as proof that either reduced panel can substitute for multimodal gait-laboratory assessment. This interpretation is consistent with recent work validating wearable inertial systems and IMU-derived gait quantification in stroke, where measurement feasibility is clear but the relationship between simpler sensor outputs and richer biomechanical organization remains only partial (35, 36). It is also aligned with integrated wearable-system studies showing that task condition and asymmetry are tightly coupled, and with pressure-sensing work indicating that clinically attractive sensing formats do not eliminate the need for thoughtful feature definition (37, 38).

The domain-level burden profile further sharpens the translational message. Gastrocnemius and knee-angle waveforms contributed prominently to both deviation and asymmetry, while pelvis angles contributed comparatively less. This pattern is clinically plausible because the most informative exported signals may be those most closely linked to propulsion timing, swing-phase knee behavior, and interlimb coordination, rather than those that are merely easy to measure. Current work with smartphone-based motion capture, wearable dual-task assessment, and sensor-augmented clinical testing also indicates that apparently simple digital measures can become far more informative when they preserve mechanistic linkage to movement structure rather than functioning only as spatiotemporal proxies (39, 40). The same lesson appears in studies showing that instrumented mobility tests change under cognitive loading and in trials where wearable-sensor gait analysis has been used to quantify treatment-related change (41, 42).

The complementary value of the deviation and asymmetry layers is equally important. Normative deviation captured how far each side departed from the able-bodied reference, whereas asymmetry captured how far the two sides diverged from one another. The higher asymmetry burden observed in sEMG-containing summaries suggests that bilateral imbalance in muscle activation may remain pronounced even when the visible sagittal waveform pattern is only moderately asymmetric. This observation resonates with current intervention literature. Meta-analyses of haptic feedback, neuromuscular electrical stimulation, and electrical-stimulation strategies increasingly emphasize that post-stroke gait change cannot be judged solely by walking speed or a single kinematic outcome (43–45). Systematic review evidence on rhythmic auditory stimulation and related wearable-supported interventions points in the same direction, namely that different technologies may influence timing, coordination, or balance through partially distinct mechanisms (46).

At the same time, the sensitivity analyses reinforce a necessary caution. The strongest stability was observed for the kinematics-only panel and for the available-case comparison, whereas sEMG-containing panels were more affected by alternate scaling and by coarse waveform compression. This does not invalidate the sEMG signal family. Instead, it indicates that some clinically attractive summaries are more representation-dependent than others, particularly when amplitude-normalized muscle activity is compared across participants and sides. Interactive telerehabilitation systems, sensor-based mobility programs, and broader wearable stroke pathways all presuppose that a reduced analytic pipeline can generate outputs stable enough for clinical interpretation outside specialized laboratories (47, 48). Our findings suggest that elements of such pipelines may be feasible, but they should not be assumed to be plug-and-play. This measured view is consistent with current narrative work on equitable wearable implementation after stroke, which argues that translational success depends not only on hardware access, but also on clear interpretation rules and transparent failure modes (49).

### 4.2 Methodological implications, limitations, and future directions

From a mechanistic standpoint, the present findings support the idea that post-stroke gait should be interpreted as a coordinated but reorganized control problem rather than as a collection of isolated deficits. Reviews of muscle-synergy applications in stroke rehabilitation and empirical studies of altered synergy structure in stiff-knee gait both emphasize that clinically visible waveforms may reflect deeper changes in neuromuscular organization (50, 51). Experimental work on cortical drive and compensatory use of the paretic limb likewise suggests that between-side differences cannot be understood solely as passive spillover from a weaker limb (52). The current asymmetry results are compatible with that literature. High gastrocnemius, semitendinosus, and knee-related asymmetry burdens imply that the exported waveform summaries retained traces of these broader control reorganizations. Targeted biofeedback trials further reinforce this point because their efficacy depends on whether the chosen signal actually captures the structure the intervention aims to change (53).

The representative waveform pattern and the domain ranking also fit with contemporary work on stiff-knee gait and late-swing knee dysfunction after stroke. Between-limb differences in peak knee flexion have recently been shown to identify clinically meaningful stiff-knee behavior, and rectus femoris transfer work has further highlighted that altered knee kinematics can coexist with broader functional consequences (54, 55). Our study does not attempt to diagnose stiff-knee gait, nor could it do so from exported spreadsheet summaries alone. Even so, the prominence of knee-angle burden and the visually delayed knee-flexion profile in the representative case support the view that knee waveform organization remains one of the most informative components of a parsimonious post-stroke gait signature.

Several limitations define the boundaries of interpretation. The current study was deliberately restricted to public spreadsheet exports rather than to the full MATLAB structures or raw trial files. This made the workflow transparent, but it also removed stride-level variability, event arrays, raw force-plate detail in the stroke cohort, and several opportunities for alternative signal preprocessing. The resulting candidate markers are summaries of already processed participant-level waveforms, not first-principles reconstructions of the original laboratory recordings. sEMG-containing panels were based on smaller complete-case samples than the kinematics-only panel, and the spreadsheet-restricted exports did not allow a participant-level demographic or clinical comparison between sEMG-complete and sEMG-incomplete stroke participants. The able-bodied reference cohort also spanned a broad adult age range, and the public export did not preserve separate control-side representations. Accordingly, the present normative framework provides a transparent broad public reference rather than an age-stratified, sex-stratified, speed-matched, or side-aware clinical normative model. Full-cycle burden summaries were intentionally simple, but post-stroke abnormalities may be concentrated in loading response, terminal stance, pre-swing, or swing; the broad-window sensitivity analysis tested this issue only coarsely and does not replace phase-specific waveform analysis. Recent scoping and systematic reviews of wearable stroke gait analysis repeatedly note that validation against gold-standard equipment remains central to trustworthy simplification (56, 57). The present study should be understood in that spirit. It proposes a reproducible intermediate workflow and a set of internally benchmarked candidate waveform-derived markers, not a validated clinical technology. It benchmarks internally within one public resource and does not validate a clinical device, establish external responsiveness, or justify replacing full gait-laboratory assessment.

Future work should extend the present framework rather than overstate it. One clear direction is to determine whether the current deviation and asymmetry summaries remain informative when broader movement tasks such as turning, dual-tasking, or home-based monitoring are introduced (57). Another is to test whether similar biomarker logic can support digital or augmented rehabilitation environments in which movement feedback is embedded in therapy rather than measured only at assessment time (58). A further step would be to connect these parsimonious summaries to real-time wearable estimation pipelines, including low-burden IMU configurations, while preserving explicit awareness of what information is lost when the multimodal laboratory reference is compressed (59). In that future pathway, the most defensible role for the present study is as a transparent intermediate layer. It demonstrates that clinically interpretable subject-level candidate gait biomarkers can be derived reproducibly from public waveform exports, while also showing that reduced panels preserve only part of the multimodal information they seek to approximate.

## 5 Conclusions

This secondary analysis of a public multimodal gait dataset showed that a spreadsheet-restricted eleven-domain waveform space can be converted into reproducible subject-level candidate waveform-derived markers of post-stroke gait. The resulting framework preserved two clinically relevant perspectives at the same time: side-specific deviation from a broad able-bodied reference and within-subject asymmetry between the paretic and non-paretic side. Both reduced signal panels retained substantial ranking information relative to the combined kinematic-sEMG internal reference panel, but neither fully reproduced its information content. Kinematics-only summaries were generally the most stable under robustness testing, whereas sEMG-containing summaries added asymmetry and muscle-timing information at the cost of greater sensitivity to alternate scoring choices and smaller complete-case samples.

These findings support a cautious translational message. Parsimonious waveform summaries may help bridge rich gait-laboratory measurement and future scalable assessment workflows, but in the present work they should be interpreted as internally benchmarked candidate markers rather than as externally validated device outputs, clinical biomarkers, or rehabilitation endpoints. The next step is to determine which reduced representations remain stable, interpretable, and clinically meaningful when tested against external outcomes, independent cohorts, phase-specific analyses, and prospective rehabilitation use.

## Data Availability

The datasets presented in this study can be found in online repositories. The names of the repository/repositories and accession number(s) can be found below: https://doi.org/10.6084/m9.figshare.c.6503791.v1.
