# Supplementary material for: Reproducible candidate kinematic-electromyographic waveform markers of post-stroke gait from public multimodal waveform exports
Source: Front Med Technol. 2026 Jul 2;8:1863908. doi: 10.3389/fmedt.2026.1863908 (PMC13373056; doi:10.3389/fmedt.2026.1863908)
Supplement: Supplementary file 1 [file Table1.docx]

**Supplementary Material 1. Full Variable Dictionary Used in the Present Analysis.** This dictionary provides the exact one-to-one mapping between the public spreadsheet exports columns and the final analytic domains retained in the present analysis. It should be read as the authoritative crosswalk for reproducible data extraction from the uploaded Excel exports.

| **Final analytic domain** | **Able-bodied spreadsheet column** | **Stroke paretic spreadsheet column** | **Stroke non-paretic spreadsheet column** | **Signal family** | **Nominal unit/scale** | **Primary reduced panel membership** | **Combined panel membership** |
| --- | --- | --- | --- | --- | --- | --- | --- |
| **Ankle angle waveform** | AnkleAngles | Pside_AnkleAngles | Nside_AnkleAngles | Sagittal kinematics | degrees | Kinematics-only | Also included in the combined panel |
| **Knee angle waveform** | KneeAngles | Pside_KneeAngles | Nside_KneeAngles | Sagittal kinematics | degrees | Kinematics-only | Also included in the combined panel |
| **Hip angle waveform** | HipAngles | Pside_HipAngles | Nside_HipAngles | Sagittal kinematics | degrees | Kinematics-only | Also included in the combined panel |
| **Pelvis angle waveform** | PelvisAngles | Pside_PelvisAngles | Nside_PelvisAngles | Sagittal kinematics | degrees | Kinematics-only | Also included in the combined panel |
| **Gastrocnemius normalized EMG waveform** | GASnorm | Pside_GASnorm | Nside_GASnorm | Normalized EMG | unitless normalized amplitude | EMG-only | Also included in the combined panel |
| **Rectus femoris normalized EMG waveform** | RFnorm | Pside_RFnorm | Nside_RFnorm | Normalized EMG | unitless normalized amplitude | EMG-only | Also included in the combined panel |
| **Vastus lateralis normalized EMG waveform** | VLnorm | Pside_VLnorm | Nside_VLnorm | Normalized EMG | unitless normalized amplitude | EMG-only | Also included in the combined panel |
| **Biceps femoris normalized EMG waveform** | BFnorm | Pside_BFnorm | Nside_BFnorm | Normalized EMG | unitless normalized amplitude | EMG-only | Also included in the combined panel |
| **Semitendinosus normalized EMG waveform** | STnorm | Pside_STnorm | Nside_STnorm | Normalized EMG | unitless normalized amplitude | EMG-only | Also included in the combined panel |
| **Tibialis anterior normalized EMG waveform** | TAnorm | Pside_TAnorm | Nside_TAnorm | Normalized EMG | unitless normalized amplitude | EMG-only | Also included in the combined panel |
| **Erector spinae normalized EMG waveform** | ERSnorm | Pside_ERSnorm | Nside_ERSnorm | Normalized EMG | unitless normalized amplitude | EMG-only | Also included in the combined panel |

***Note.*** *The able-bodied export stores one participant-level waveform per domain without separate left-right columns, whereas the stroke export preserves paretic and non-paretic waveforms separately.* **Acronyms.** EMG = surface electromyography; GAS = gastrocnemius; RF = rectus femoris; VL = vastus lateralis; BF = biceps femoris; ST = semitendinosus; TA = tibialis anterior; ERS = erector spinae.
