# Supplementary material for: Reproducible candidate kinematic-electromyographic waveform markers of post-stroke gait from public multimodal waveform exports
Source: Front Med Technol. 2026 Jul 2;8:1863908. doi: 10.3389/fmedt.2026.1863908 (PMC13373056; doi:10.3389/fmedt.2026.1863908)
Supplement: Supplementary file 2 [file Table2.docx]

**Supplementary Material 2. Domain-Level Availability and Quality Checks.** Counts in this table refer to complete 1001-point waveforms in the public spreadsheet exports. The paired-asymmetry column indicates whether both paretic and non-paretic waveforms were simultaneously available for a given domain, which is the requirement for within-stroke asymmetry analyses.

| **Domain** | **Signal family** | **Able-bodied complete waveforms** | **Stroke paretic complete waveforms** | **Stroke non-paretic complete waveforms** | **Paired asymmetry complete waveforms** | **Primary analytic status** | **Quality note** |
| --- | --- | --- | --- | --- | --- | --- | --- |
| **AnkleAngles** | Sagittal kinematics | 138/138 (100.0%) | 50/50 (100.0%) | 50/50 (100.0%) | 50/50 (100.0%) | Included in the primary analysis | No missing waveform points; complete coverage in all exported subjects. |
| **KneeAngles** | Sagittal kinematics | 138/138 (100.0%) | 50/50 (100.0%) | 50/50 (100.0%) | 50/50 (100.0%) | Included in the primary analysis | No missing waveform points; complete coverage in all exported subjects. |
| **HipAngles** | Sagittal kinematics | 138/138 (100.0%) | 50/50 (100.0%) | 50/50 (100.0%) | 50/50 (100.0%) | Included in the primary analysis | No missing waveform points; complete coverage in all exported subjects. |
| **PelvisAngles** | Sagittal kinematics | 138/138 (100.0%) | 50/50 (100.0%) | 50/50 (100.0%) | 50/50 (100.0%) | Included in the primary analysis | No missing waveform points; complete coverage in all exported subjects. |
| **GASnorm** | Normalized EMG | 109/138 (79.0%) | 43/50 (86.0%) | 44/50 (88.0%) | 43/50 (86.0%) | Included in the primary analysis | All missingness was waveform-level all-or-none; nonmissing waveforms had complete 1001-point coverage. |
| **RFnorm** | Normalized EMG | 108/138 (78.3%) | 43/50 (86.0%) | 44/50 (88.0%) | 43/50 (86.0%) | Included in the primary analysis | All missingness was waveform-level all-or-none; nonmissing waveforms had complete 1001-point coverage. |
| **VLnorm** | Normalized EMG | 109/138 (79.0%) | 43/50 (86.0%) | 44/50 (88.0%) | 43/50 (86.0%) | Included in the primary analysis | All missingness was waveform-level all-or-none; nonmissing waveforms had complete 1001-point coverage. |
| **BFnorm** | Normalized EMG | 106/138 (76.8%) | 43/50 (86.0%) | 44/50 (88.0%) | 43/50 (86.0%) | Included in the primary analysis | All missingness was waveform-level all-or-none; nonmissing waveforms had complete 1001-point coverage. |
| **STnorm** | Normalized EMG | 108/138 (78.3%) | 43/50 (86.0%) | 44/50 (88.0%) | 43/50 (86.0%) | Included in the primary analysis | All missingness was waveform-level all-or-none; nonmissing waveforms had complete 1001-point coverage. |
| **TAnorm** | Normalized EMG | 107/138 (77.5%) | 43/50 (86.0%) | 44/50 (88.0%) | 43/50 (86.0%) | Included in the primary analysis | All missingness was waveform-level all-or-none; nonmissing waveforms had complete 1001-point coverage. |
| **ERSnorm** | Normalized EMG | 106/138 (76.8%) | 46/50 (92.0%) | 46/50 (92.0%) | 46/50 (92.0%) | Included in the primary analysis | All missingness was waveform-level all-or-none; nonmissing waveforms had complete 1001-point coverage. ERS showed slightly higher availability than the other stroke EMG domains. |

***Note.*** *No partial within-waveform missingness was detected in either uploaded spreadsheet. Missingness therefore operated at the complete-waveform level in the current exports.*

**Acronyms.** EMG = surface electromyography; ERS = erector spinae.
