# Supplementary material for: Reproducible candidate kinematic-electromyographic waveform markers of post-stroke gait from public multimodal waveform exports
Source: Front Med Technol. 2026 Jul 2;8:1863908. doi: 10.3389/fmedt.2026.1863908 (PMC13373056; doi:10.3389/fmedt.2026.1863908)
Supplement: Supplementary file 3 [file Table3.docx]

**Supplementary Material 3. Full Mathematical Definitions of Digital Biomarkers.** This supplement formalizes the quantities used in the present MedTech workflow. The notation is intentionally aligned with the public spreadsheet exports so that the formulas can be reproduced directly from the accessible 1001-point waveforms without reconstructing the full MATLAB stride structures. For a given domain d and a given normalized time sample t, x_d,s(t) denotes the observed stroke waveform for side s, with s equal to the paretic side P or non-paretic side N. The pointwise able-bodied reference mean and standard deviation are denoted by μ_d(t) and σ_d(t), respectively. The pointwise able-bodied median and the MAD-derived robust dispersion are denoted by m_d(t) and σ*_d(t). All summaries are computed only on finite waveform samples and all present analyses were restricted to the exported 1001-point waveforms available in the uploaded spreadsheets.

The primary deviation panel score for a given participant-side combination was defined as the arithmetic mean of domain-level MASD values across the domains belonging to that panel. The primary asymmetry panel score for a given participant was defined as the arithmetic mean of domain-level MASA values across the domains belonging to that panel. The primary complete-case workflow required all domains of a panel to be present. The combined available-case sensitivity analysis relaxed that rule only for the combined panel and accepted participants when all four shared kinematic domains were present, while averaging across whatever subset of the eleven combined domains was available.

Robust normative dispersion replaced μ_d(t) and σ_d(t) with m_d(t) and σ*_d(t). The ERS-exclusion sensitivity removed ERSnorm from EMG-containing panels. The broad-window representation reduced each waveform to five coarse windows spanning approximately 0–20%, 20–40%, 40–60%, 60–80%, and 80–100% of the normalized cycle before recomputing the primary deviation burden summary. Amplitude-normalized asymmetry centered and scaled each limb waveform by its own within-waveform mean and standard deviation before forming the side-difference curve.

**Notation**

| **Symbol** | **Definition** |
| --- | --- |
| **x_d,s(t)** | Observed stroke waveform for domain d, side s, and normalized time sample t |
| **μ_d(t)** | Pointwise able-bodied reference mean for domain d |
| **σ_d(t)** | Pointwise able-bodied reference standard deviation for domain d |
| **m_d(t)** | Pointwise able-bodied reference median for domain d |
| **σ*_d(t)** | Pointwise robust dispersion, computed as 1.4826 × MAD at each time sample with SD fallback only when the robust estimate is numerically negligible |
| **CT** | Temporal center of mass of a normalized EMG waveform, expressed as percent gait cycle |

**Normative deviation biomarkers**

| **Normative-deviation biomarker** | **Formula** | **Interpretive use in the present workflow** |
| --- | --- | --- |
| **MASD_d,s** | mean_t \|(x_d,s(t) − μ_d(t)) / σ_d(t)\| | Primary deviation burden score used to anchor panel benchmarking |
| **RMSD_d,s** | sqrt(mean_t [((x_d,s(t) − μ_d(t)) / σ_d(t))^2]) | Secondary deviation summary with greater sensitivity to large departures |
| **PASD_d,s** | max_t \|(x_d,s(t) − μ_d(t)) / σ_d(t)\| | Largest standardized departure observed within the normalized cycle |
| **TONC95_d,s** | 100 × mean_t I[x_d,s(t) outside μ_d(t) ± 1.96σ_d(t)] | Percent of the cycle spent outside the reference corridor |
| **NWC_d,s** | corr(x_d,s(t), μ_d(t)) | Waveform-shape similarity to the normative reference |

**Within-stroke asymmetry biomarkers.**

| **Within-stroke asymmetry biomarker** | **Formula** | **Interpretive use in the present workflow** |
| --- | --- | --- |
| **MASA_d** | mean_t \|(x_d,P(t) − x_d,N(t)) / σ_d(t)\| | Primary asymmetry burden score used to anchor asymmetry panel summaries |
| **RMSA_d** | sqrt(mean_t [((x_d,P(t) − x_d,N(t)) / σ_d(t))^2]) | Asymmetry summary that emphasizes larger interlimb differences |
| **SWC_d** | corr(x_d,P(t), x_d,N(t)) | Waveform-shape similarity between the two sides |
| **AECTA_m** | \|CT_m,P − CT_m,N\| | Absolute difference in EMG activity centroid timing for muscle m, expressed as percent gait cycle |

**Sensitivity-analysis implementation details.**

| **Sensitivity scenario** | **Implementation in the current workflow** | **Primary rationale** |
| --- | --- | --- |
| **Robust normative dispersion** | m_d(t) = median_t(control values); σ*_d(t) = 1.4826 × MAD with SD fallback when needed | Tests dependence on non-robust scaling |
| **ERS-exclusion** | Remove ERSnorm from EMG-only and combined panels and recompute the same panel-score formula | Tests trunk-channel dependence |
| **Broad-window representation** | Replace each 1001-point waveform by five window means before computing MASD | Tests dependence on local waveform detail |
| **Combined available-case rule** | Accept the combined panel when all four shared kinematic domains are present and compute the mean over available combined domains | Quantifies the cost of strict multimodal completeness |
| **Amplitude-normalized asymmetry** | Center and scale each side waveform within subject before computing MASA | Tests whether asymmetry ranking is dominated by amplitude |

**Acronyms.** MAD = median absolute deviation; MASD = mean absolute standardized deviation; RMSD = root mean square standardized deviation; PASD = peak absolute standardized deviation; TONC95 = time outside the 95% normative corridor; NWC = normative waveform correlation; MASA = mean absolute standardized asymmetry; RMSA = root mean square standardized asymmetry; SWC = side waveform correlation; AECTA = absolute EMG centroid timing asymmetry; EMG = surface electromyography; ERS = erector spinae.
