# Supplementary material for: Reproducible candidate kinematic-electromyographic waveform markers of post-stroke gait from public multimodal waveform exports
Source: Front Med Technol. 2026 Jul 2;8:1863908. doi: 10.3389/fmedt.2026.1863908 (PMC13373056; doi:10.3389/fmedt.2026.1863908)
Supplement: Supplementary file 4 [file Table4.docx]

**Supplementary Material 4. Full Results of Sensitivity Analyses.** Panel A reports sensitivity results for deviation-based panel scores, while Panel B reports the corresponding results for asymmetry-based panel scores. Spearman rho close to 1 indicates stable participant ranking relative to the primary workflow, whereas larger score differences or higher quartile-reclassification percentages indicate greater sensitivity to the alternative specification.

**Panel A. Deviation-score sensitivity results**

| **Sensitivity scenario** | **Panel** | **Side** | **Primary n** | **Sensitivity n** | **Overlap n** | **Spearman rho** | **Median absolute score difference** | **90th percentile absolute score difference** | **Quartile reclassification (%)** | **Added cases** |
| --- | --- | --- | --- | --- | --- | --- | --- | --- | --- | --- |
| **Robust normative dispersion** | Combined kinematic-EMG | Non-paretic | 44 | 44 | 44 | 0.9815 | 0.2799 | 0.4187 | 61.4 | 0 |
| **Robust normative dispersion** | Combined kinematic-EMG | Paretic | 43 | 43 | 43 | 0.9906 | 0.2719 | 0.4339 | 55.8 | 0 |
| **Robust normative dispersion** | EMG-only | Non-paretic | 44 | 44 | 44 | 0.9794 | 0.4067 | 0.5429 | 63.6 | 0 |
| **Robust normative dispersion** | EMG-only | Paretic | 43 | 43 | 43 | 0.9876 | 0.3969 | 0.636 | 60.5 | 0 |
| **Robust normative dispersion** | Kinematics-only | Non-paretic | 50 | 50 | 50 | 0.9946 | 0.0821 | 0.1753 | 14.0 | 0 |
| **Robust normative dispersion** | Kinematics-only | Paretic | 50 | 50 | 50 | 0.9964 | 0.0779 | 0.1537 | 20.0 | 0 |
| **Broad-window representation** | Combined kinematic-EMG | Non-paretic | 44 | 44 | 44 | 0.9787 | 0.1827 | 0.2424 | 56.8 | 0 |
| **Broad-window representation** | Combined kinematic-EMG | Paretic | 43 | 43 | 43 | 0.9873 | 0.1928 | 0.2937 | 46.5 | 0 |
| **Broad-window representation** | EMG-only | Non-paretic | 44 | 44 | 44 | 0.9731 | 0.2334 | 0.3324 | 70.5 | 0 |
| **Broad-window representation** | EMG-only | Paretic | 43 | 43 | 43 | 0.9869 | 0.2447 | 0.399 | 62.8 | 0 |
| **Broad-window representation** | Kinematics-only | Non-paretic | 50 | 50 | 50 | 0.994 | 0.0616 | 0.12 | 16.0 | 0 |
| **Broad-window representation** | Kinematics-only | Paretic | 50 | 50 | 50 | 0.9909 | 0.0668 | 0.1524 | 6.0 | 0 |
| **ERS-exclusion analysis** | Combined kinematic-EMG | Non-paretic | 44 | 44 | 44 | 0.9645 | 0.0306 | 0.0991 | 18.2 | 0 |
| **ERS-exclusion analysis** | Combined kinematic-EMG | Paretic | 43 | 43 | 43 | 0.9905 | 0.0482 | 0.1156 | 11.6 | 0 |
| **ERS-exclusion analysis** | EMG-only | Non-paretic | 44 | 44 | 44 | 0.9474 | 0.0475 | 0.17 | 31.8 | 0 |
| **ERS-exclusion analysis** | EMG-only | Paretic | 43 | 43 | 43 | 0.9704 | 0.0698 | 0.1877 | 23.3 | 0 |
| **Complete-case versus available-case** | Combined kinematic-EMG | Non-paretic | 44 | 50 | 44 | 1.0 | 0.0 | 0.0 | 0.0 | 6 |
| **Complete-case versus available-case** | Combined kinematic-EMG | Paretic | 43 | 50 | 43 | 1.0 | 0.0 | 0.0 | 0.0 | 7 |

**Panel B. Asymmetry-score sensitivity results**

| **Sensitivity scenario** | **Panel** | **Primary n** | **Sensitivity n** | **Overlap n** | **Spearman rho** | **Median absolute score difference** | **90th percentile absolute score difference** | **Quartile reclassification (%)** | **Added cases** |
| --- | --- | --- | --- | --- | --- | --- | --- | --- | --- |
| **Amplitude-normalized asymmetry** | Combined kinematic-EMG | 43 | 43 | 43 | 0.8375 | 0.4711 | 0.7575 | 74.4 | 0 |
| **Amplitude-normalized asymmetry** | EMG-only | 43 | 43 | 43 | 0.7477 | 0.6096 | 0.9891 | 74.4 | 0 |
| **Amplitude-normalized asymmetry** | Kinematics-only | 50 | 50 | 50 | 0.7779 | 0.1722 | 0.8001 | 58.0 | 0 |
| **ERS-exclusion analysis** | Combined kinematic-EMG | 43 | 43 | 43 | 0.9881 | 0.0335 | 0.0795 | 14.0 | 0 |
| **ERS-exclusion analysis** | EMG-only | 43 | 43 | 43 | 0.9585 | 0.0435 | 0.1443 | 27.9 | 0 |
| **Complete-case versus available-case** | Combined kinematic-EMG | 43 | 50 | 43 | 1.0 | 0.0 | 0.0 | 0.0 | 7 |

***Note.*** *The complete-case versus available-case comparison only added cases in the combined panel. It did not change overlapping participant scores because the primary score for overlapping participants was already defined on the full domain set.*

**Acronyms.** ERS = erector spinae; EMG = surface electromyography.
